# Genetic Diversity and Population Structure in *Polygonum cespitosum*: Insights to an Ongoing Plant Invasion

**DOI:** 10.1371/journal.pone.0093217

**Published:** 2014-04-02

**Authors:** Silvia Matesanz, Kathryn E. Theiss, Kent E. Holsinger, Sonia E. Sultan

**Affiliations:** 1 Área de Biodiversidad y Conservación. Departamento de Biología y Geología, Universidad Rey Juan Carlos, Móstoles, Spain; 2 Biology Department, Willamette University, Salem, Oregon, United States of America; 3 Department of Ecology & Evolutionary Biology, University of Connecticut, Storrs, Connecticut, United States of America; 4 Biology Department, Wesleyan University, Middletown, Connecticut, United States of America; University of Bordeaux, France

## Abstract

Molecular markers can help elucidate how neutral evolutionary forces and introduction history contribute to genetic variation in invaders. We examined genetic diversity, population structure and colonization patterns in the invasive *Polygonum cespitosum*, a highly selfing, tetraploid Asian annual introduced to North America. We used nine diploidized polymorphic microsatellite markers to study 16 populations in the introduced range (northeastern North America), via the analyses of 516 individuals, and asked the following questions: 1) Do populations have differing levels of within-population genetic diversity? 2) Do populations form distinct genetic clusters? 3) Does population structure reflect either geographic distances or habitat similarities? We found low heterozygosity in all populations, consistent with the selfing mating system of *P. cespitosum*. Despite the high selfing levels, we found substantial genetic variation within and among *P. cespitosum* populations, based on the percentage of polymorphic loci, allelic richness, and expected heterozygosity. Inferences from individual assignment tests (Bayesian clustering) and pairwise *F*_ST_ values indicated high among-population differentiation, which indicates that the effects of gene flow are limited relative to those of genetic drift, probably due to the high selfing rates and the limited seed dispersal ability of *P. cespitosum*. Population structure did not reflect a pattern of isolation by distance nor was it related to habitat similarities. Rather, population structure appears to be the result of the random movement of propagules across the introduced range, possibly associated with human dispersal. Furthermore, the high population differentiation, genetic diversity, and fine-scale genetic structure (populations founded by individuals from different genetic sources) in the introduced range suggest that multiple introductions to this region may have occurred. High genetic diversity may further contribute to the invasive success of *P. cespitosum* in its introduced range.

## Introduction

Genetic variation can be substantially altered when species are introduced into new ranges. The amount of genetic variation and its distribution within and among populations in the new range is determined by the number of introductions, the diversity of the founders, mating system and other life-history traits, and post-introduction processes such as genetic drift, gene flow, and selection [1]–[5].

As a result of the introduction and invasion processes, genetic variation is often dramatically reduced, since populations in the introduced range are usually established by a small number of founders representing only a fraction of the genetic diversity present in the native range [3], [6]–[7]. Loss of genetic variation can have important implications for the invasion dynamics of introduced species, since it may limit a species' ability to adapt to the new conditions [8]. Although strong founder effects and population bottlenecks have often been observed in introduced-range populations of invasive species [5], [9]–[11], similar or even higher genetic variation in the introduced compared to the native range has also been found [4], [12]–[15]. Multiple introductions can reduce bottleneck effects, especially if introduction events come from genetically differentiated native populations. Neutral molecular markers such as microsatellites can help elucidate introduction history and its effects on genetic variation and population structure in an introduced range, which in turn can provide insights into colonization patterns, potential for evolution, and invasion success [3], [7], [13], [14], [16].

Life history traits such as mating system can also be a strong determinant of both within-population variation and population structure. Self-compatibility and/or apomictic reproduction have long been recognized as a key characteristic of ideal weeds [17], [18], and indeed, many invasive species have uniparental reproduction (see [19]). Compared to outcrossing species, populations of selfing or apomictic species that are derived from only a few founders generally show low levels of within-population variation, high homozygosity, and strong population structure resulting from low gene flow and increased genetic drift [1], [6], [13]. The amount and distribution of genetic variation in the introduced range in selfing species will thus depend on the relative effects of founder effects, colonization events and selfing rates.

*Polygonum* (s.l.) *cespitosum* Blume ( = *Persicaria cespitosa*, [20]) is a highly selfing, tetraploid, annual species native to eastern Asia, from China to Japan and Southeast Asia [20]–[23]. It was introduced to North America in the early 20^th^ century and has been reported in most states in the eastern and central United States [24]. Recently it has been catalogued as invasive in the northeastern United States (New England states) due to its rapid, aggressive spread in this region [21] where it was first reported circa 1930 [25]. In the native range, and initially in North America, *P. cespitosum* was mostly restricted to moist, shaded habitats such as forest understories [23], [26]. Over the last 15–20 years, however, it has begun to colonize open, drier sites in its introduced range, where it forms dense stands and shows greater performance –higher individual reproductive success and higher population abundances– than in low-light sites (Horgan-Kobelski, Matesanz, and Sultan, in revision). However, it is not known whether this rapid, ongoing range expansion in the introduced range is caused by the preferential movement of a subset of genotypes to the new habitats or by random colonization events by multiple genotypes.

In this study, we examined genetic diversity and population structure in the introduced range of *P. cespitosum* using microsatellite markers. We studied a set of 16 populations that represent the current ecological distribution of *P. cespitosum* in northeastern North America. Although the species is present in a large area of the United States, our study focuses on populations from the portion of the introduced range where the species has been catalogued as invasive [21], [27]. Furthermore, to gain insights into colonization patterns of new habitats in the introduced range, we used detailed environmental characterization of the study populations and related it to population genetic structure. Specifically, we addressed the following questions: 1) Do populations have differing levels of within-population genetic diversity? 2) Do populations form distinct genetic clusters? 3) Does population structure reflect geographic distances or habitat similarities?

## Methods

### Population sampling

**Figure 1:**
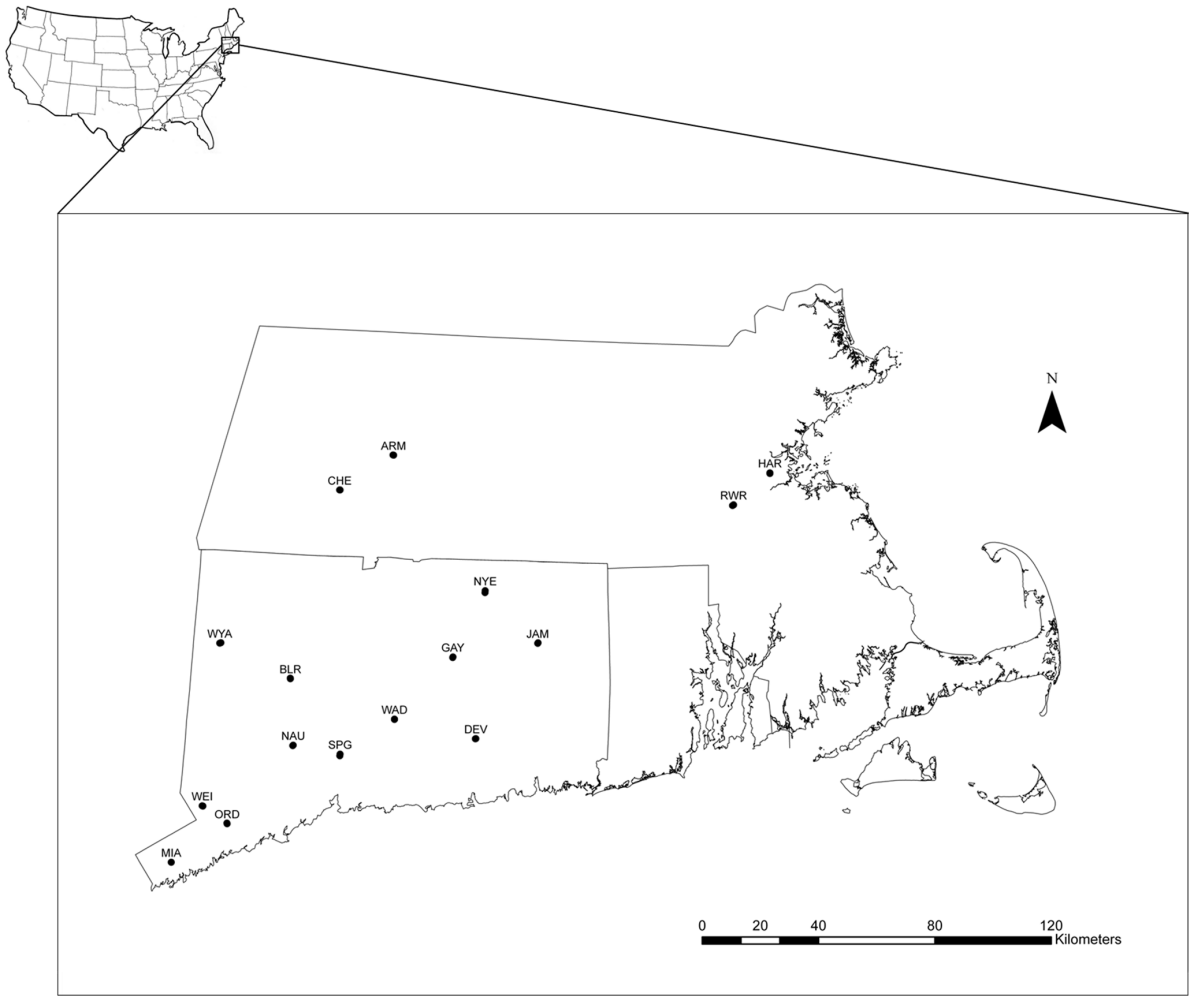
Location of sampled sites in North America. Letter codes correspond to the population codes listed in Table 1.

We used field [23] and herbarium records from the George Safford Torrey Herbarium, University of Connecticut, to identify *Polygonum cespitosum* populations in northeastern North America, where this species has been recently classified as invasive [21]. In October 2008, 16 well-established *Polygonum cespitosum* populations in northeastern North America (Connecticut and Massachusetts, USA) were selected (see [28] for details on population selection; Fig. 1; see Table 1 for geographic coordinates). In each population, we collected achenes (single-seeded fruit) from 22–45 individuals along linear transects at intervals of approximately one 1 m. Populations were characterized with respect to light and soil moisture availability twice during the growing season of the species (early July and September 2009; Table 1). Light availability was quantified using hemispherical canopy photography; 15 hemispherical pictures were taken in each population. Soil moisture was calculated gravimetrically by extracting 10 soil cores (at two depths, 0–10 cm and 20–30 cm) from two transects covering the spatial extent of each population (see [28] and Horgan-Kobelski, Matesanz and Sultan, in revision, for a detailed description of the measurements protocols and environmental data for each population). Local site conditions are related both to the performance of individual plants and to the performance of populations and provide a proxy of long-term, site-specific light and water availability (Horgan-Kobelski, Matesanz and Sultan, in revision). No specific permits were required for the described field studies, as the locations were not privately-owned or protected in any way and there was no involvement of endangered or protected species.

**Table 1:** Population code, location, geographical coordinates and habitat type for the 16 *Polygonum cespitosum* populations from the introduced range used in this study.

Population Code	Introduced population location	Geographical coordinates	Type of habitat	GSF	Soil moisture
ARM	Arch Road, Leeds, MA	42°21′13″N, 72°41′39″W	Roadside	0.44	46.56
BRL	Black Rock State Park, Thomaston, CT	41°39′24″N, 73°06′18″W	Trailhead and forest edge	0.18	52.22
CHE	Chester-Blandford State Forest, Chester, MA	42°14′35″N, 72°54′56″W	Trailhead	0.19	92.55
DEV	Devils Hopyard State Park, East Haddam, CT	41°28′42″N, 72°20′30″W	Roadside	0.20	65.09
GAY	Gay City State Park, Hebron, CT	41°43′47″N, 72°26′20″W	Forest trail	0.13	57.99
HAR	Harvard Arnold Arboretum, Jamaica Plain, MA	42°18′08″N, 71°07′27″W	Lowland clearing	0.41	141.19
JAM	James Goodwin State Forest, Hampton, CT	41°46′40″N, 72°05′12″W	Forest horse trail	0.14	71.93
MIA	Mianus River State Park, Stamford, CT	41°04′51″N, 73°34′50″W	Clearing by trailhead and parking lot	0.26	88.85
NAU	Naugatuck Forest, Oxford, CT	41°26′58″N, 73°05′34″W	Roadside	—	—
NYE	Nye Holman State Forest, Tolland, CT	41°52′55″N, 72°18′27″W	Forest path and meadow	0.31	67.94
ORD	Katherine Ordway Preserve, Weston, CT	41°12′19″N, 73°21′24″W	Trailhead and lawn edge	0.26	57.19
RWR	Rocky Wood Reservation, Medfield, MA	42°12′13″N, 71°16′49″W	Forest horse trail	—	—
SPG	Sleeping Giant State Park, Hamden, CT	41°25′15″N, 72°53′55″W	Trailhead and picnic area	0.15	78.05
WAD	Wadsworth Estate, Middletown, CT	41°32′07″N, 72°40′33″W	Forest horse trail and clearing	0.27	80.40
WEI	Weir Farm, Wilton, CT	41°15′23″N, 73°27′22″W	Roadside	0.31	57.42
WYA	Wyantenock State Forest, Kent, CT	41°45′47″N, 73°23′52″W	Forest trail	0.18	77.86

Site means for light availability (global site factor, GSF) and soil moisture (% of field capacity) are also shown. Soil moisture levels ≥100% means flooded soil. See text for details.

In March 2009, field-collected achenes were grown in a glasshouse as described in [29], and three to four leaves were collected from each individual and immediately frozen at −80°C for later DNA extraction. In total, we sampled 516 individuals from 16 populations.

### DNA extraction and microsatellite markers

Total genomic DNA was extracted from 100 mg of frozen leaf tissue using the DNeasy Plant Mini kit (Qiagen, Valencia, CA, USA), and its concentration and purity was quantified on a NanoDrop spectrophotometer (NanoDrop Products, Wilmington, DE, USA). DNA concentration ranged from 20 to 100 ng/μl. We genotyped each sample at seven microsatellite loci known to be polymorphic across the species (described in [30]): Poce1, Poce3, Poce11, Poce15, Poce20, Poce26 and Poce28. We performed two multiplexing PCR reactions with dye-labeled primers (Applied Biosystems, Foster City, CA, USA and Eurofins MWG Operon, Huntsville, Alabama, USA) using the Type-it Microsatellite PCR kit (Qiagen, Valencia, CA, USA): one with primers Poce3, Poce11, Poce15 and Poce20, and a second one with primers Poce1 and Poce 28. The multiplex PCR reactions contained 2.75 μL of RNase-free water, 6.25 μL of Master Mix, 1.25 μL of the primer mix (each primer at 2 μM), 1.25 μL of Q solution and 1 μL of DNA. An Applied Biosystems 2720 Thermal Cycler (Applied Biosystems, Foster City, CA, USA) was used with the following settings: 5 min at 95°C, 28 cycles of 30 s at 95°C, 90 s at 57°C and 30 s at 72°C, and a final cycle of 30 min at 60°C. A separate PCR was performed with primer Poce26, containing 8.65 μL of RNase-free water, 1.25 μL of GeneAmp Buffer (with MgCl_2_) with 0.1 μL of AmpliTaq Gold DNA polymerase (Applied Biosystems, Foster City, CA, USA), 0.5 μL of dye-labeled forward primer (10 μM), 0.5 μL of reverse primer (10 μM), 0.5 μL of premixed dNTP (2.5 mM each, Epicentre Biotechnologies, Madison, WI, USA) and 1.5 μL of DNA. Thermocycling consisted of a touchdown thermal cycling program [31] encompassing a 10°C span of annealing temperatures ranging between 65°C and 55°C. Amplification success of each reaction was checked by running 4 μL of PCR product of 15 haphazardly selected samples per 96-well plate on a 1% agarose gel stained with Sybr Green gel stain (Cambrex Biosciences, Rockland, ME, USA). PCR products (1 μL) were mixed with 9.2 μL of HI-DI formamide and 0.3 μL of GeneScan 600 LIZ size standard (Applied Biosystems) and analyzed on an Applied Biosystems 3730xl DNA Analyzer at the Life Sciences Core Laboratories Center at Cornell University (http://cores.lifesciences.cornell.edu/brcinfo/).

### Microsatellite fragment scoring

DNA fragments were scored manually using GeneMarker (Softgenetics, State College, PA, USA). *P. cespitosum* is tetraploid both in its native and introduced ranges, but it is not known whether it is an auto- or allotetraploid [20]. Despite its polyploidy, five of the markers (Poce1, Poce3, Poce11, Poce15 and Poce20) behaved as diploids, consistently amplifying one or two alleles per individual. However, the two remaining markers (Poce26 and Poce28) amplified up to four alleles per individual. For Poce26, the segregation patterns of individual alleles allowed us to identify what appeared to be two diploidized homeologous loci [32]. For Poce28, assignment of alleles was more complex due to the relatively high number of individuals in which only one allele was observed. Therefore, we used two different coding schemes for data from this marker. Every individual (with the exception of ARM23) contained either the 305 or 319 allele. Therefore in the first coding scheme, these two alleles were assigned to the first homeologous locus and all other alleles were assigned to the second locus. Individuals for which only one allele was observed were scored as being homozygous at the first locus and having missing data at the second locus, while individuals with two observed alleles were scored as homozygous for the appropriate allele at each locus. The single individual that had four observable alleles (HAR11) was scored as being heterozygous at both homeologous loci (305/319 and 361/365). In the second coding scheme, we did not restrict alleles 305 and 319 to the first locus. Therefore, individuals with one allele were scored as homozygous at both loci, and the individuals that amplified two alleles were scored as being homozygous at the first locus and having one copy of the appropriate allele and missing data in the second allele of the second locus.

In order to confirm the assignment of alleles for marker Poce26 and to provide additional insight into allelic relations at Poce28, PCR products of several individuals were cloned using the TOPO TA Cloning kit (Invitrogen, Grand Island, NY, USA) and sequenced with the BigDye Terminator v3.1 Cycle Sequencing Kit (Applied Biosystems). For marker Poce26, we identified a six bp indel in the flanking region surrounding the tetramer repeat, whose presence or absence corresponded to the assignment of alleles described above. For Poce28, all size differences were attributable to differences in repeat number. Apparently, the homeologous copies have not yet diverged. We present results using the first coding scheme for this locus because it minimizes the amount of missing data. Analyses using the second coding scheme (not presented) produced very similar results. Furthermore, analyses leaving out data from both Poce26 and Poce28 also produced very similar results.

In summary, we scored all individuals for nine diploidized loci. Fewer than 1% of all individuals were missing data. Because a moderate number of individuals had fragments that were inconsistent with whole repeat numbers, we scored microsatellite alleles as Mendelian alleles, not as repeat counts.

### Data analysis

#### Genetic diversity within populations

We calculated the following genetic diversity indices for each population using Arlequin v. 3.11 [33] and Genalex v. 6.41 [34]: *P*, proportion of polymorphic loci; *A*, mean number of alleles per locus (allele richness); *A_e_*, mean number of effective alleles (1/Σ*p*_i_^2^, where *p*_i_ is the frequency of the *i* ^th^ allele for the population), *H_o_*, observed heterozygosity (number of heterozygotes/N, where N is the number of individuals per population); *H_e_*, unbiased expected heterozygosity ((2N/(2N-1)) * (1-Σ*p*_i_^2^)); *F*_IS_, inbreeding coefficient (1-(*H_o_*/*H_e_*)); the number of private alleles and the number of multilocus genotypes. To obtain a conservative estimate of the number of multilocus genotypes, we ignored the loci with missing data.

Allelic richness after correcting for unequal sample sizes (rarefaction), *A_rare_*, was inferred using the rarefaction method implemented in Hp-Rare [35]. Rarefaction is a statistical technique to deal with unequal sample sizes so that the number of alleles can be compared among samples. As the smallest sample analyzed consisted of 22 individuals (SPG population), the number of sampled alleles per locus was set to 44 for this calculation. Deviation from Hardy-Weinberg equilibrium was evaluated within each population with the Markov Chain Monte Carlo approximation (dememorization = 10000, batches = 100, iterations per batch = 10000) of Fisher's exact test implemented in Genepop v. 4.1 (Rousset, 2008). In order to test for a reduction in effective population size linked to bottleneck or founder events, heterozygosity tests were performed in BOTTLENECK 1.2.02 [36] to compare the estimates of expected heterozygosity based on allele frequencies and on the number of alleles and sample size. When a population experiences a bottleneck, the number of alleles decreases faster than heterozygosity, resulting in an apparent excess of heterozygosity [36]. Wilcoxon tests with 2000 iterations were used under the stepwise-mutation model (SMM), the infinite allele model (IAM), and the two-phase model with 5% of multi-step mutations, as recommended for microsatellites. Finally, Pearson correlation coefficients were computed between the sample size in each population and each genetic diversity index.

#### Population genetic structure

To determine population differentiation we computed pairwise *F*_ST_ [37] with *P*-values for each pair of populations (90000 permutations) using Arlequin. We used a conservative Bonferroni correction to account for multiple comparisons. To test for isolation by distance (IBD), a Mantel test [38] between the matrix of pairwise genetic differentiation between populations (*F*_ST_), and the matrix of geographical (Euclidean) distances between populations was performed with 9999 permutations using Arlequin. The analysis was repeated using the matrix of logarithm of the distance between populations [39], and similar results were obtained.

In order to gain insight into the patterns of colonization in the introduced range, Mantel tests were performed (with 9999 permutations) between the matrix of pairwise genetic differentiation between populations (*F*_ST_), and the matrix of differentiation in light availability (Euclidean distance in GSF) and soil moisture (Euclidean distance in percentage of field capacity) between populations. Finding a significant correlation between the matrix of genetic differentiation and those of differentiation in light and soil moisture availability would be interpreted as non-random establishment of genotypes in different habitats (i.e. new habitats are colonized by a subset of genotypes instead of a random sample). These tests were performed for the 14 populations for which environmental data were available (Table 1).

We used a model-based Bayesian clustering method implemented in the program STRUCTURE v. 2.3 [40] to assign individuals to unique genetic clusters. STRUCTURE assumes a model in which there are *K* populations (where *K* is unknown), each of which is characterized by a set of allele frequencies at each locus. Individuals are then probabilistically assigned to one or more clusters. The membership of each individual in a cluster is estimated as a coefficient that ranges from zero to one, with one indicating full membership in a cluster. We performed 10 independent runs for each value of *K* ranging from one to 16 using a burn-in period of 10^5^ iterations followed by a sample of 10^6^ iterations. We used the default parameters of the program to allow population admixture and correlated allele frequency across populations [41]. We used HARVESTER [42] to extract the relevant data from STRUCTURE results files and to generate CLUMPP input files. We then used CLUMPP v. 1.2.2 [43] to combine results from the 10 runs at each *K*., using the Greedy option for *K* values of three to five and the LargeKGreedy option for *K* values greater than five. Membership in clusters was visualized using the program DISTRUCT v. 1.1 [44].

To determine the number of clusters most appropriate for the interpretation of our data, we first calculated the mean log probability of the data for each *K*, and determined the value of *K* for which this probability was the highest. Second, we calculated Δ*K* following the method described in [45]. Δ*K* is a quantity based on the rate of change in the log probability of the data between successive *K* values.

## Results

### Genetic variation within populations

In the 516 individuals analyzed, a total of 88 alleles were identified for the nine microsatellite loci, an average of 9.8 alleles per locus. The average number of alleles per locus ranged from three (Poce28–1) to 23 (Poce20).

Genetic diversity varied substantially across populations. The percentage of polymorphic loci per population was high in all populations (≥60%), with the exception of WEI, where all loci were monomorphic. The average number of alleles observed per locus, *A*, ranged from one to four. Rarefaction of the number of alleles per locus to a standardized sample rendered almost identical results (range 1–3.96). The number of multilocus genotypes varied across populations from one to 17, but was ≤10 for most populations (12 out of 16). We found a total of 27 private alleles, present in 10 of the 16 populations. The number of private alleles per population ranged from one to five (Table 2).

**Table 2:** Genetic diversity indices of the 16 *Polygonum cespitosum* populations using nine microsatellite loci.

Population code	*N*	*P*	*A*	*A_rare_*	*A_e_*	*H_o_*	*H_e_*	*F*_IS_	Nb. of private alleles	Nb. of genotypes
ARM	34	100	3.22	3.15	1.42	0.003	0.260	0.987	4	8
BLR	34	100	2.67	2.57	1.87	0.000	0.435	1.000	0	4
CHE	35	100	3.33	3.01	1.29	0.023	0.228	0.875	3	9
DEV	29	100	2.67	2.58	1.73	0.020	0.347	0.944	5	7
GAY	31	55.56	1.67	1.59	1.04	0.004	0.039	0.909	0	3
HAR	33	88.89	3.67	3.44	1.84	0.089	0.371	0.756	2	17
JAM	35	100	3.11	2.79	1.25	0.016	0.172	0.908	2	6
MIA	33	100	4.00	3.96	3.18	0.003	0.646	0.994	3	12
NAU	25	77.78	3.67	3.59	2.23	0.027	0.411	0.935	1	9
NYE	45	88.89	3.00	2.88	1.76	0.010	0.385	0.974	2	16
ORD	32	100	3.56	3.34	2.03	0.070	0.456	0.856	0	17
RWR	28	88.89	2.11	2.09	1.12	0.000	0.108	1.000	2	4
SPG	22	88.89	2.78	2.78	1.92	0.000	0.445	1.000	0	6
WAD	33	88.89	3.11	2.91	1.40	0.003	0.260	0.987	3	10
WEI	34	0	1.00	1.00	1.00	0.000	0.000	---	0	1
WYA	33	77.78	2.11	2.01	1.11	0.007	0.099	0.933	0	5
Overall	516	84.723	2.85	2.64	1.64	0.017	0.291	0.937	27	8.375

*N*, number of individuals sampled; *P*, proportion of polymorphic loci; *A*, mean number of alleles per locus, *A_rare_*, mean number of alleles with rarefaction; *A_e_*, mean number of effective alleles, *H_o_*, observed heterozygosity; *H_e_*, expected heterozygosity; *F*_IS_, inbreeding coefficient; number of private alleles and number of multilocus genotypes. See text for details on statistics.

Expected heterozygosity ranged from zero (WEI) to 0.646 (MIA, Table 2). Observed heterozygosity was lower than 0.1 in all populations. The fixation index (*F*_IS_) varied among populations from 0.756 to 1 (Table 2); all polymorphic populations showed a significant deficiency of heterozygotes at all loci (*P*<0.001 across loci and populations). Genetic diversity within populations was not related to sample size (*P*>0.22 for all comparisons).

Under the infinite allele model, four populations deviated significantly from mutation-drift equilibrium (Appendix S1), but only one population (MIA) showed evidence of a recent bottleneck under all models (Appendix S1).

### Population structure

Population pairwise *F*_ST_ values were highly significant and generally very high (see Appendix S2), ranging from low, 0.094 between populations ARM and NYE to 0.976 between GAY and WEI. Pairwise *F*_ST_ were higher than 0.5 in ≥60% of the comparisons.

**Figure 2:**
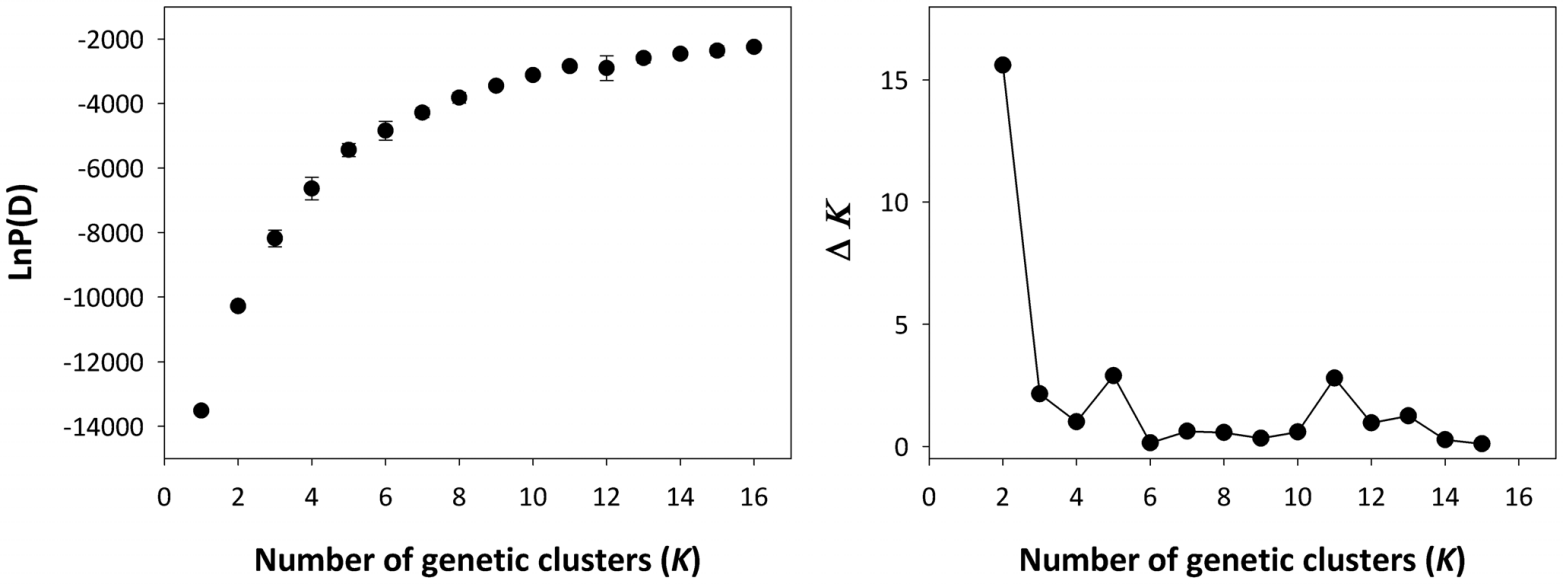
Left: Mean log probability of the data for the 10 Structure runs at each *K*. Error bars are standard deviations; Right: Δ*K*, rate of change in the log probability of data between successive *K* values, as described by Evanno et al. [45].

**Figure 3:**
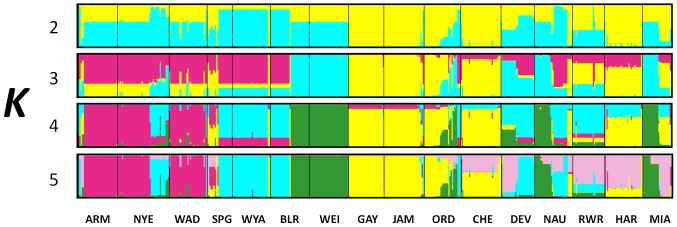
Population structure inferred by Bayesian cluster analyses (Structure) for 516 *Polygonum cespitosum* individuals from 16 populations. Results for *K* (number of clusters) ranging from 2 to 16 are shown. Each individual (grouped by population) is represented by a vertical bar. The proportion of the bar in each of *K* colors corresponds to the average posterior likelihood that the individual is assigned to the cluster indicated by that color. Populations are separated by black lines, and are arranged according to the observed clusters.

A strict interpretation of our results using the method of Evanno et al. [45] would suggest that two genetic clusters are sufficient for interpretation of our data (*K* = 2; Fig. 2). We choose to focus instead on the results with *K* = 5 for several reasons: 1) there is a secondary large peak in Δ*K* at *K* = 5, 2) the rate at which the mean estimated log probability of the data [LnP(D)] increases slows markedly at *K* = 5 (Fig. 2) *K* = 5 is more consistent with the evidence for high levels of among-population differentiation revealed in pairwise *F*_ST_ comparisons, and 4) at higher values of *K*, no additional genetic clusters characteristic of individual sampling locations are identified. We present results for *K* = 2–5 to provide a comprehensive understanding of the structure in our data (Fig. 3; see Appendix S3 for results for *K* = 2–16).

In the *K* = 5 solution, most populations were composed of individuals belonging to a single genetic cluster (e.g. WEI, GAY, JAM and WYA). In some instances, individuals from multiple populations were assigned to the same genetic cluster: one cluster included ARM, NYE and WAD (pink, Fig. 3), a second cluster (blue) included SPG and WYA, and the yellow cluster included GAY, JAM and ORD (Fig. 3). Conversely, some populations contained individuals assigned to different genetic clusters. For instance, BLR contained individuals assigned to two different clusters (dark green and blue), NYE contained individuals assigned to the pink and blue clusters and MIA contained individuals assigned to the green and pink clusters. Finally, the individuals of some populations were not completely assigned to any genetic clusters (CHE, RWR and HAR).

We detected no significant isolation by distance (IBD) between populations, either using the matrix of linear Euclidean distances (*R*_M_ = 0.073; *P* = 0.311) or the log of the distances (*R*_M_ = 0.087; *P* = 0.271). Similarly, we did not find any evidence that populations more similar to one another along either environmental axis (light availability and soil moisture) were also more genetically similar (*R*_M_ = 0.077; *P* = 0.295 and *R*_M_ = −0.164; *P* = 0.212 for light availability and soil moisture, respectively).

## Discussion

In this study, we assessed genetic variation in 16 populations of the selfing invasive species *Polygonum cespitosum*, to understand the dynamics of the species' invasion and its population structure, and to explore whether the rapid range expansion observed in this species is caused by the preferential movement of a subset of genotypes to the new habitats or by multiple, random colonization events.

All populations showed large heterozygote deficiencies at every locus. Low heterozygosity is consistent with the selfing mating system of *P. cespitosum*, and has been reported in several other self-compatible invasive species ([4], [5], [9], [13]; reviewed in [6]). In highly selfing species, a higher proportion of the genetic variation tends to be distributed among rather than within populations (see [6], [12], [13]). In the case of *P. cespitosum*, the majority of the genetic variation was indeed found across populations, as shown by the high *F*_ST_ values observed, and Bayesian assignment tests showed that populations were grouped in a few very distinct genetic clusters.

However, the strong population structure in the studied populations did not reflect isolation by distance, i.e. closer populations were not genetically more similar than populations farther apart. Indeed, individuals from geographically distant populations were in some cases assigned to the same genetic cluster (see [4], [13], [46] for similar results). A pattern of isolation by distance emerges when populations are likely to be founded by close neighbors or when they share genetic material via the distance-limited dispersal of pollen or seeds [47]. Our results suggest that the effects of gene flow among populations are limited relative to those of genetic drift, possibly due to the combination of high selfing rates and limited seed dispersal ability in *P. cespitosum* [23], as has also been shown in other studies with highly-selfing invasives [13].

Our failure to detect a pattern of isolation by distance could be explained if populations preferentially established into habitats similar to those from which they came, but we found no evidence of such a pattern. We detected no association between the distance between populations on environmental axes – light and soil moisture availability– and the genetic distance between them. Initially, *P. cespitosum* was mainly restricted to shaded, moist habitats in northeastern North America, but recently the species has expanded to open habitats characterized by high light availability and potential soil moisture deficits (Horgan-Kobelski, Matesanz and Sultan, in revision). The lack of a detectable association between genetic and environmental factors suggests that recent colonization of open sites is occurring in the form of multiple independent events, as opposed to the spread of a similarly adapted subset of genotypes (see [48] and references therein). These results agree with a recent study showing that this newly invasive species consists of highly plastic, generalist populations that can successfully establish in environmentally diverse sites [28].

Population structure in the introduced range is consistent with random establishment of genotypes in different areas, possibly mediated by human dispersal. *P. cespitosum* occurs in highly disturbed sites such as roadsides and forest paths, and large populations are often found in public parks and forests where human presence is high. This situation has likely fostered the movement of propagules across the introduced range. Individual populations might be founded by relatively few individuals, but the source of those individuals bears little relationship to the geographical or ecological distance from the site where new populations are established. Such a pattern of colonization would lead strongly differentiated populations with apparently random degrees of relatedness. Our results concur with other studies showing highly differentiated populations in the introduced range [4], [9], [13], [49], and highlight the role of human-mediated dispersal as well as the idiosyncrasy of the invasion process [50], [51].

Alongside high among-population differentiation, populations of primarily-selfing introduced species are expected to exhibit low amounts of genetic diversity, particularly if founded by only a few propagules introduced from a single source population [6], [52]. However, we found substantial genetic variation within and among *P. cespitosum* populations, as shown by the average number of alleles and the expected heterozygosity. These results suggest that *P. cespitosum* may have not undergone a genetic bottleneck in the introduced range. Indeed, only one population showed evidence of having experienced a recent bottleneck under the two-phase model recommended for microsatellite data. This possibility is further supported by the fact that a sample of four native Asian *P. cespitosum* populations had similar (or even lower) genetic variation (e.g. within-population allelic richness and expected heterozygosity) than the studied introduced-range populations (see details in Appendix S4). In a recent review, Dlugosch and Parker [3] showed that significant losses of both allelic richness and heterozygosity in introduced-range populations are frequent (see also [5], [12]–[14]). Our results agree with a few case studies where, rather than losses of genetic variation, increased molecular variation was found in the introduced range, such as for the invasive plant species *Bromus tectorum* and *Phalaris arundinacea* and the lizard *Anolis sagrei* [12], [15], [53].It is possible that the limited number of native-range populations and/or the smaller genotypic samples from those populations (Appendix S4) is insufficient to provide a robust test. Extensive sampling of native populations might reveal further differences between ranges in levels of genetic variation.

We have two arguments that suggest that multiple independent introductions of *P. cespitosum* may have occurred in North America. First, our limited sample of genetic variation in Asian populations suggests that a single introduction from one native-range population is unlikely because of the great diversity and the high among-population differentiation found in North American populations (see [1], [11], [16] for studies where low population differentiation is interpreted as indicative of few native sources). Second, we found that some populations include individuals belonging to two distinct clusters (e.g. DEV, BLR, and NYE populations, Fig. 3), suggesting that they were founded from multiple sources. Multiple introductions appear to be common for invasive species [4], [13], [14], [54], and can reduce the expected loss of genetic variation due to introduction in selfing species [5], [6], as seems to be the case in *P. cespitosum*.

Our study shows that despite high levels of inbreeding, *P. cespitosum* exhibits considerable levels of genetic variation in the introduced range, likely due to the occurrence of multiple past introductions. Variation in neutral markers is often a poor indicator of variation in quantitative traits ([3], [55]–[57] but see [58], [59]), but in highly selfing species the entire genome is inherited as a unit, and variation in neutral markers is likely to be associated with variation in quantitative traits [60], [61]. Thus, our results suggest that high evolutionary potential in fitness-related traits may be present in the introduced range. This is supported by recent studies showing quantitative genetic variation as well as rapid adaptive evolution within introduced-range populations of *P. cespitosum* [8], [62]. Another intriguing finding in this system is that certain introduced-range *P. cespitosum* populations contain exceptionally high-performance generalist genotypes likely to contribute to future invasiveness [29]. Further comparisons with Asian populations will be needed to determine if these genotypes are the evolutionary results of population mixing following multiple introductions, as has been found in other species [63], [64]. Furthermore, contrasting levels of genetic variation across populations in the introduced range suggests that invasion trajectories and future invasion potential may differ among populations. Subsequent admixture or intraspecific hybridization between previously isolated genotypes could further increase genetic variation and contribute to the evolution of novel genotypes in *P. cespitosum* [29], [65].

## Supporting Information

Appendix S1 **Probabilities from Wilcoxon sign-rank tests for heterozygosity excess (population bottlenecks) in 16 populations of** ***Polygonum cespitosum*** **under the infinite allele (IAM), the two-phase (TPM) and stepwise mutation models (SMM).**
(DOCX)

Appendix S2 **Pairwise** ***F*** **_ST_ values for all population comparisons.** All values are significant (except for two comparisons, underlined) after Bonferroni correction for multiple comparisons (corrected *P*-value ≈0.0003).
(DOCX)

Appendix S3 **Population structure inferred by Bayesian cluster analyses for 516** ***Polygonum cespitosum*** **individuals from 16 populations (** ***K*** **= 2–16).**
(DOCX)

Appendix S4 **Genetic diversity and population structure of 4** ***Polygonum cespitosum*** **populations from the native range.**
(DOCX)
