# Supplementary material for: Genetic Diversity and Population Structure in Polygonum cespitosum: Insights to an Ongoing Plant Invasion
Source: PLoS One. 2014 Apr 2;9(4):e93217. doi: 10.1371/journal.pone.0093217 (PMC3973574; doi:10.1371/journal.pone.0093217)
Supplement: Appendix S1 — Probabilities from Wilcoxon sign-rank tests for heterozygosity excess (population bottlenecks) in 16 populations of Polygonum cespitosum under the infinite allele (IAM), the two-phase (TPM) and stepwise mutation models (SMM). (DOCX) [file pone.0093217.s001.docx]

**Appendix S1.** Probabilities from Wilcoxon sign-rank tests for heterozygosity excess (population bottlenecks) in 16 populations of *Polygonum cespitosum* under the infinite allele (IAM), the two-phase (TPM) and stepwise mutation models (SMM).

| Population | IAM | TPM | SMM |
| --- | --- | --- | --- |
| ARM | 0.998 | 1.000 | 1.000 |
| BLR | **0.024** | 0.213 | 0.213 |
| CHE | 0.993 | 0.997 | 0.998 |
| DEV | 0.213 | 0.787 | 0.820 |
| GAY | 1.000 | 1.000 | 1.000 |
| HAR | 0.578 | 0.986 | 0.986 |
| JAM | 0.999 | 1.000 | 1.000 |
| MIA | **0.001** | **0.001** | **0.001** |
| NAU | 0.289 | 0.766 | 0.852 |
| NYE | **0.020** | 0.527 | 0.629 |
| ORD | 0.125 | 0.715 | 0.850 |
| RWR | 1.000 | 1.000 | 1.000 |
| SPG | **0.006** | 0.191 | 0.191 |
| WAD | 0.973 | 0.994 | 0.994 |
| WEI | 1.000 | 1.000 | 1.000 |
| WYA | 1.000 | 1.000 | 1.000 |
