# Supplementary material for: Genetic Diversity and Population Structure in Polygonum cespitosum: Insights to an Ongoing Plant Invasion
Source: PLoS One. 2014 Apr 2;9(4):e93217. doi: 10.1371/journal.pone.0093217 (PMC3973574; doi:10.1371/journal.pone.0093217)
Supplement: Appendix S2 — Pairwise F ST values for all population comparisons. All values are significant (except for two comparisons, underlined) after Bonferroni correction for multiple comparisons (corrected P-value ≈0.0003). (DOCX) [file pone.0093217.s002.docx]

**Appendix S2.** Pairwise *F*_ST_ values for all population comparisons. All values are significant (except for two comparisons, underlined) after Bonferroni correction for multiple comparisons (corrected *P*-value = 0.0003).

|  | **ARM** | **BLR** | **CHE** | **DEV** | **GAY** | **HAR** | **JAM** | **MIA** | **NAU** | **NYE** | **ORD** | **RWR** | **SPG** | **WAD** | **WEI** |
| --- | --- | --- | --- | --- | --- | --- | --- | --- | --- | --- | --- | --- | --- | --- | --- |
| **BLR** | 0.558 |  |  |  |  |  |  |  |  |  |  |  |  |  |  |
| **CHE** | 0.711 | 0.578 |  |  |  |  |  |  |  |  |  |  |  |  |  |
| **DEV** | 0.652 | 0.339 | 0.643 |  |  |  |  |  |  |  |  |  |  |  |  |
| **GAY** | 0.784 | 0.686 | 0.752 | 0.796 |  |  |  |  |  |  |  |  |  |  |  |
| **HAR** | 0.541 | 0.455 | 0.542 | 0.515 | 0.606 |  |  |  |  |  |  |  |  |  |  |
| **JAM** | 0.701 | 0.612 | 0.645 | 0.716 | 0.137 | 0.515 |  |  |  |  |  |  |  |  |  |
| **MIA** | 0.453 | 0.185 | 0.455 | 0.308 | 0.581 | 0.373 | 0.508 |  |  |  |  |  |  |  |  |
| **NAU** | 0.587 | 0.324 | 0.528 | 0.380 | 0.694 | 0.442 | 0.609 | 0.175 |  |  |  |  |  |  |  |
| **NYE** | 0.094 | 0.434 | 0.635 | 0.531 | 0.688 | 0.456 | 0.619 | 0.363 | 0.502 |  |  |  |  |  |  |
| **ORD** | 0.514 | 0.342 | 0.411 | 0.457 | 0.274 | 0.312 | 0.212 | 0.223 | 0.289 | 0.436 |  |  |  |  |  |
| **RWR** | 0.766 | 0.612 | 0.789 | 0.625 | 0.899 | 0.620 | 0.800 | 0.514 | 0.627 | 0.647 | 0.583 |  |  |  |  |
| **SPG** | 0.502 | 0.194 | 0.575 | 0.393 | 0.685 | 0.352 | 0.574 | 0.213 | 0.424 | 0.362 | 0.299 | 0.637 |  |  |  |
| **WAD** | 0.273 | 0.565 | 0.723 | 0.668 | 0.796 | 0.556 | 0.712 | 0.453 | 0.599 | 0.233 | 0.525 | 0.772 | 0.504 |  |  |
| **WEI** | 0.841 | 0.579 | 0.863 | 0.779 | 0.976 | 0.789 | 0.895 | 0.481 | 0.492 | 0.723 | 0.648 | 0.940 | 0.788 | 0.846 |  |
| **WYA** | 0.745 | 0.373 | 0.811 | 0.633 | 0.904 | 0.641 | 0.819 | 0.485 | 0.701 | 0.594 | 0.601 | 0.848 | 0.295 | 0.751 | 0.943 |
