# Supplementary material for: Genetic Diversity and Population Structure in Polygonum cespitosum: Insights to an Ongoing Plant Invasion
Source: PLoS One. 2014 Apr 2;9(4):e93217. doi: 10.1371/journal.pone.0093217 (PMC3973574; doi:10.1371/journal.pone.0093217)

**Appendix S3.** Population structure inferred by Bayesian cluster analyses (Structure) for 516 *Polygonum cespitosum* individuals from 16 populations. Results for *K* (number of clusters) ranging from 2 to 16 are shown. Each individual (grouped by population) is represented by a vertical bar. The proportion of the bar in each of *K* colors corresponds to the average posterior likelihood that the individual is assigned to the cluster indicated by that color. Populations are separated by black lines, and are arranged according to the observed clusters.


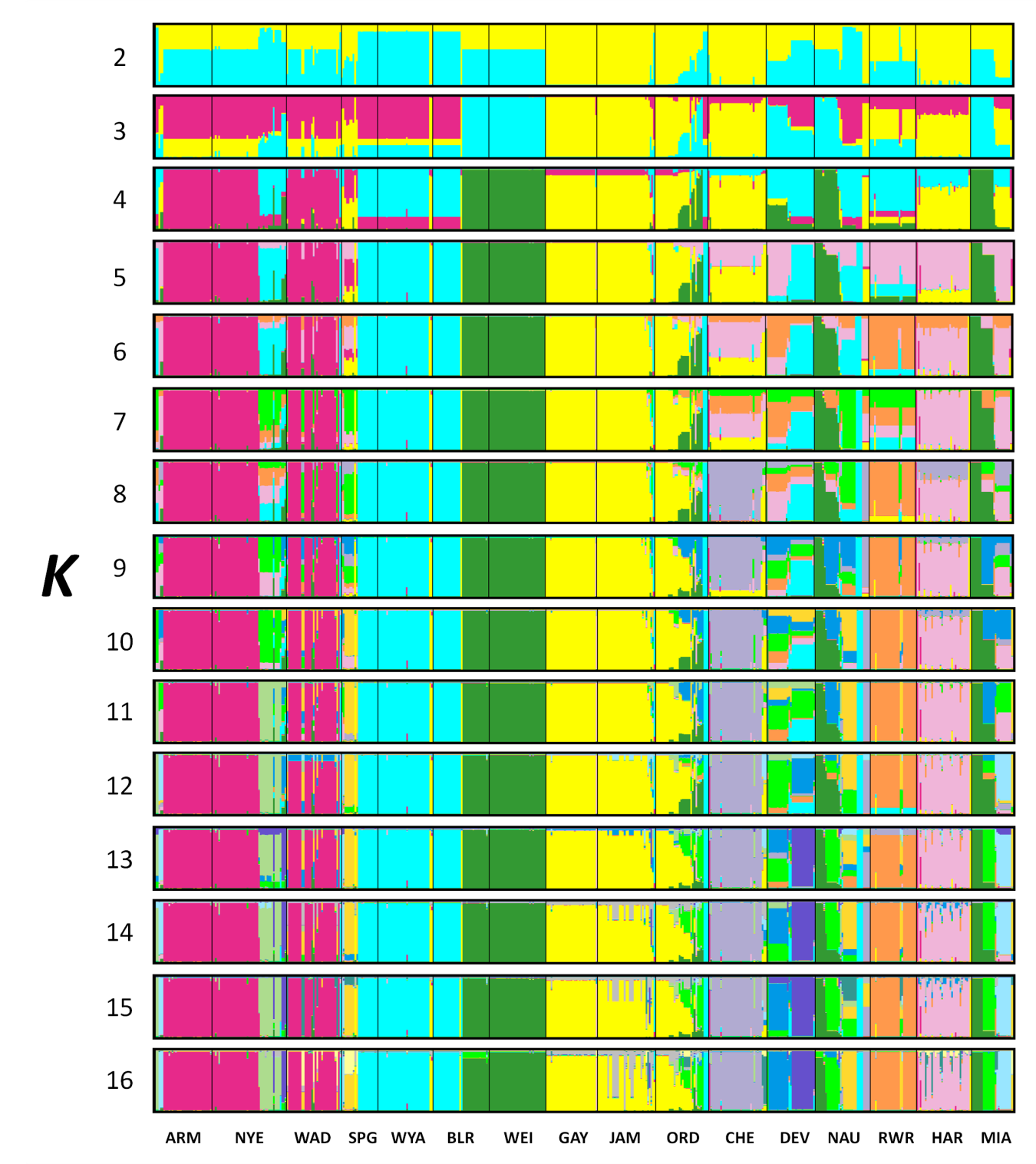

Supplement: Appendix S3 — Population structure inferred by Bayesian cluster analyses for 516 Polygonum cespitosum individuals from 16 populations ( K = 2–16). (DOCX) [file pone.0093217.s003.docx]
