# Supplementary material for: Genetic Diversity and Population Structure in Polygonum cespitosum: Insights to an Ongoing Plant Invasion
Source: PLoS One. 2014 Apr 2;9(4):e93217. doi: 10.1371/journal.pone.0093217 (PMC3973574; doi:10.1371/journal.pone.0093217)
Supplement: Appendix S4 — Genetic diversity and population structure of 4 Polygonum cespitosum populations from the native range. (DOCX) [file pone.0093217.s004.docx]

**Appendix S4**. **Genetic diversity and population structure of 4 *Polygonum cespitosum* populations from the native range.**

We selected 4 populations from the native range (Japan and South Korea, Table 1 and Figure 1), and collected achenes (single-seeded fruit) from 18-25 individuals along linear transects at intervals of approximately 1 m.

| **Code** | **Population location** | **Geographical coordinates** | **Type of habitat** |
| --- | --- | --- | --- |
| JPB | Inage, Chiba Prefecture, Japan | 35º38’08’’N,  140º05’02’’E | Forest edge and adjacent meadow |
| JPA | Toneunga Park, Nagareyama, Chiba Prefecture, Japan | 35º54’60’’N,  139°54’16’’E | Forest trail |
| UTK | Botanic garden, University of Tokyo, Japan | 35º40’30’’N,  139°44’46’’E | Forest trail |
| KOR | Hwajeop-ri, Byeolnae-myeon, Namyangju-si, Gyeonggi-do, South Korea | 37º38’57’’N,  127º06’10’’E | Pear orchards |

**Table 1**. Population code, location, and geographical coordinates for the 4 *Polygonum cespitosum* populations from the native range used in this study.


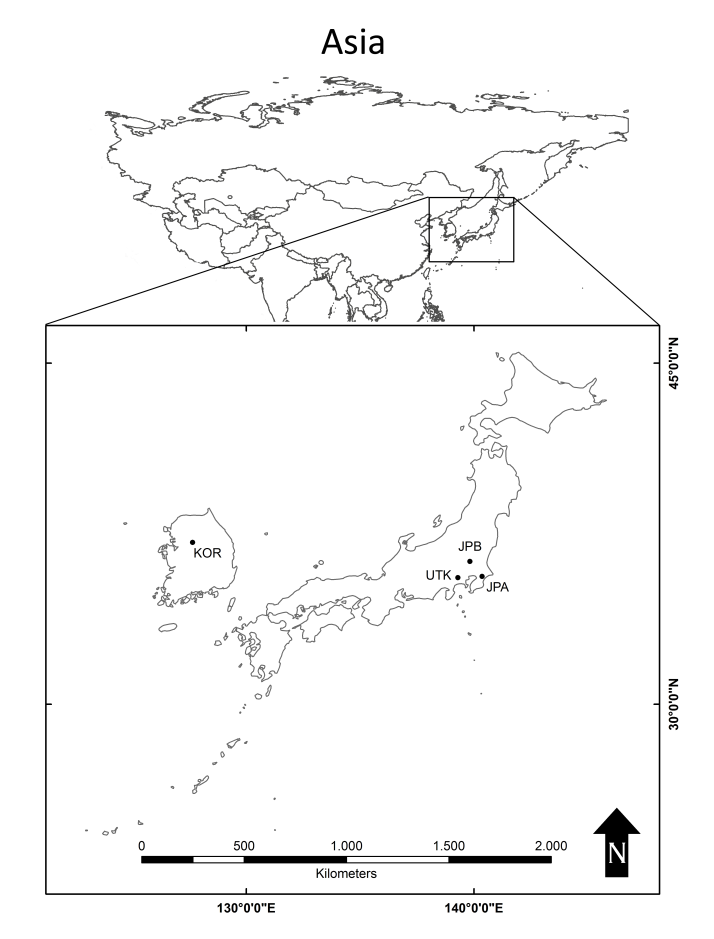


**Figure 1.** Location of sampled sites in the native range.

| **Population code** | ***N*** | ***P*** | ***A*** | ***A_rare_*** | ***A_e_*** | ***H_o_*** | ***H_e_*** | ***F*_IS_** | **Private alleles** | **Nr. genotypes** |
| --- | --- | --- | --- | --- | --- | --- | --- | --- | --- | --- |
|  |  |  |  |  |  |  |  |  |  |  |
| JPA | 25 | 100 | 2.000 | 1.926 | 1.083 | 0.000 | 0.078 | 1.000 | 0 | 2 |
| JPB | 23 | 22.22 | 1.222 | 1.217 | 1.102 | 0.000 | 0.061 | 1.000 | 0 | 3 |
| UTK | 18 | 88.89 | 2.667 | 2.657 | 1.793 | 0.006 | 0.391 | 0.983 | 3 | 4 |
| KOR | 20 | 44.44 | 2.000 | 1.975 | 1.302 | 0.044 | 0.180 | 0.758 | 1 | 8 |
| **Overall** | 86 | 63.888 | 1.972 | 1.946 | 1.320 | 0.013 | 0.178 | 0.935 | 5 | 4.250 |

**Table 2**. Genetic diversity indices of the 4 native-range *Polygonum cespitosum* populations using nine microsatellite loci. See main text for details on statistics.

Statistical comparisons of several genetic diversity indices (*P*, *A*, *A*_rare_, *A*_e_ and *H*_e_) between native and introduced-range populations were performed using non-parametric Kruskal-Wallis analysis of variance in Statistica 8.0 (Tulsa, OK USA). These comparisons were repeated using jackknife resampling, a method that accounts for the imbalance in the number of populations sampled from both ranges by recomputing the statistic estimate leaving out one observation at a time (Hill and Lewicki, 2005). Since results from both sets of analyses were virtually identical, only Kruskal-Wallis results are reported.

The percentage of polymorphic loci per population was indistinguishable in introduced-range and native populations (Kruskal-Wallis *P* = 0.196). The average number of alleles observed per locus, *A*, was higher in introduced-range populations (average = 2.85) than in native populations (average = 1.97; *P* = 0.037). Similarly, the number of alleles per locus rarified to a standardized sample was marginally higher in introduced-range than native populations (2.65 vs. 1.95 in introduced-range and native-populations, respectively; *P* = 0.072), as was the number of multilocus genotypes (8.37 vs. 4.25 in introduced-range and native-populations, respectively; *P* = 0.088). The mean number of effective alleles per population was similar in introduced-range and native populations (1.64 vs. 1.38, *P* = 0.392).
